# Inhibitory Effects of Dimethyllirioresinol, Epimagnolin A, Eudesmin, Fargesin, and Magnolin on Cytochrome P450 Enzyme Activities in Human Liver Microsomes

**DOI:** 10.3390/ijms18050952

**Published:** 2017-05-01

**Authors:** Ju-Hyun Kim, Soon-Sang Kwon, Hyeon-Uk Jeong, Hye Suk Lee

**Affiliations:** Drug Metabolism and Bioanalysis Laboratory, College of Pharmacy, The Catholic University of Korea, Bucheon 420-743, Korea; jhyunkim@catholic.ac.kr (J.-H.K.); zuzutnseo@naver.com (S.-S.K.); wjd1375@hanmail.net (H.-U.J.)

**Keywords:** dimethyllirioresinol, epimagnolin A, eudesmin, fargesin, magnolin, human liver microsomes, cytochrome P450 inhibition

## Abstract

Magnolin, epimagnolin A, dimethyllirioresinol, eudesmin, and fargesin are pharmacologically active tetrahydrofurofuranoid lignans found in Flos Magnoliae. The inhibitory potentials of dimethyllirioresinol, epimagnolin A, eudesmin, fargesin, and magnolin on eight major human cytochrome P450 (CYP) enzyme activities in human liver microsomes were evaluated using liquid chromatography-tandem mass spectrometry to determine the inhibition mechanisms and inhibition potency. Fargesin inhibited CYP2C9-catalyzed diclofenac 4′-hydroxylation with a *K*_i_ value of 16.3 μM, and it exhibited mechanism-based inhibition of CYP2C19-catalyzed [*S*]-mephenytoin 4′-hydroxylation (*K*_i_, 3.7 μM; *k*_inact_, 0.102 min^−1^), CYP2C8-catalyzed amodiaquine *N*-deethylation (*K*_i_, 10.7 μM; *k*_inact_, 0.082 min^−1^), and CYP3A4-catalyzed midazolam 1′-hydroxylation (*K*_i_, 23.0 μM; *k*_inact_, 0.050 min^−1^) in human liver microsomes. Fargesin negligibly inhibited CYP1A2-catalyzed phenacetin *O*-deethylation, CYP2A6-catalyzed coumarin 7-hydroxylation, CYP2B6-catalyzed bupropion hydroxylation, and CYP2D6-catalyzed bufuralol 1′-hydroxylation at 100 μM in human liver microsomes. Dimethyllirioresinol weakly inhibited CYP2C19 and CYP2C8 with IC_50_ values of 55.1 and 85.0 μM, respectively, without inhibition of CYP1A2, CYP2A6, CYP2B6, CYP2C9, CYP2D6, and CYP3A4 activities at 100 μM. Epimagnolin A, eudesmin, and magnolin showed no the reversible and time-dependent inhibition of eight major CYP activities at 100 μM in human liver microsomes. These in vitro results suggest that it is necessary to investigate the potentials of in vivo fargesin-drug interaction with CYP2C8, CYP2C9, CYP2C19, and CYP3A4 substrates.

## 1. Introduction

**Figure 1:**
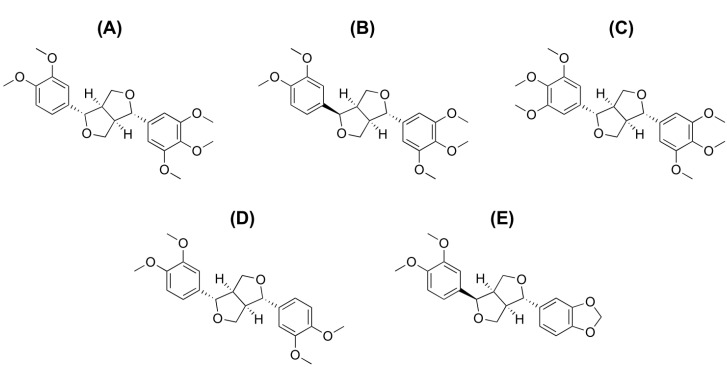
Chemical structures of (**A**) magnolin; (**B**) epimagnolin A; (**C**) dimethyllirioresinol; (**D**) eudesmin; and (**E**) fargesin.

Magnolin, epimagnolin A, dimethyllirioresinol, eudesmin, and fargesin (Figure 1) are the pharmacologically active tetrahydrofurofuranoid lignans found in Flos Magnoliae, *Aristolochia elegans* rhizomes, and *Zanthoxylum armatum* DC. [1,2,3,4,5]. Magnolin, epimagnolin A, dimethyllirioresinol, eudesmin, and fargesin exhibit various biological activities, including anti-inflammatory activity [6,7,8,9,10], 5-lipoxygenase inhibitory activity [6], antimycobacterial activity [11], and the inhibition of tumor growth and cancer-catalyzed bone destruction [12]. Fargesin exhibits additional biological activities, including β1-adrenergic receptor antagonistic and cardioprotective effects [13], stimulation of basal glucose uptake and glucose transporter-4 translocation in muscle cells [14], treatment of dyslipidemia and hyperglycemia in high-fat diet-induced obese mice via activation of Akt and 5′-adenosine monophosphate-activated protein kinase in white adipose tissue [15], and antihypertensive effects in 2K1C hypertensive rats [16]. Magnolin also inhibits cancer cell migration, invasion, and growth [17,18,19] and ameliorates contrast-induced nephropathy via antioxidation and antiapoptosis in rats [20].

Herbal drugs (e.g., *Hypericum perforatum*, *Ginkgo biloba*, *Camellia sinensis*, *Glycyrrhiza glabra*, *Allium sativum*, Rhizoma Coptidis, and Fructus Silybi) and their constituents cause herb–drug interactions via the induction or inhibition of major drug-metabolizing enzymes, cytochrome P450 (CYP) and result in the toxicity and therapeutic failure of various concomitant drugs [21,22,23,24,25,26,27,28,29,30,31]. For the prediction of herb-drug interaction, it is necessary to investigate the in vitro inhibitory effects of herb drugs and the constituents on major human CYP enzyme activities. In vitro inhibitory effects of the pharmacologically active lignans such as aschantin [32], honokiol [33], machilin A [34], phyllantin, hypophyllantin [35], and podophyllotoxin [36] on CYP enzymes have been reported. However, there are no reports on the in vitro and in vivo inhibitory effects of the bioactive tetrahydrofurofuranoid lignans such as dimethyllirioresinol, epimagnolin A, eudesmin, fargesin, and magnolin on human CYP enzymes.

In the present study, the in vitro inhibition potency and inhibition mechanisms of dimethyllirioresinol, epimagnolin A, eudesmin, fargesin, and magnolin on 8 major human CYP (CYPs 1A2, 2A6, 2B6, 2C8, 2C9, 2C19, 2D6, and 3A4) activities in pooled human liver microsomes were evaluated to decide the performance of in vivo drug interaction studies of dimethyllirioresinol, epimagnolin A, eudesmin, fargesin, and magnolin.

## 2. Results

**Figure 2:**
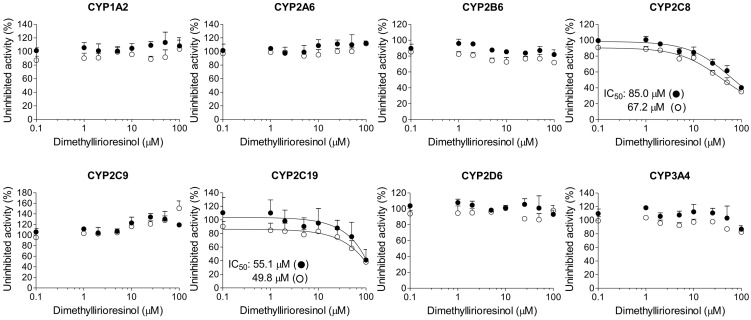
Inhibitory effects of dimethyllirioresinol on CYP1A2-mediated phenacetin *O*-deethylation, CYP2A6-mediated coumarin 7-hydroxylation, CYP2B6-mediated bupropion hydroxylation, CYP2C8-mediated amodiaquine *N*-deethylation, CYP2C9-mediated diclofenac 4′-hydroxylation, CYP2C19-mediated [*S*]-mephenytoin 4′-hydroxylation, CYP2D6-mediated bufuralol 1′-hydroxylation, and CYP3A4-mediated midazolam 1′-hydroxylation in pooled human liver microsomes. ○: Pre-incubation of liver microsomes with dimethyllirioresinol and reduced β-nicotinamide adenine dinucleotide phosphate (NADPH) for 30 min at 37 °C and ●: No pre-incubation. Data represent the average ± standard deviation (SD, *n* = 3).

The reversible and time-dependent inhibitory potencials (IC_50_ values) of dimethyllirioresinol, epimagnolin A, eudesmin, fargesin, and magnolin on 8 major human CYP enzymes were investigated in human liver microsomes. Dimethyllirioresinol weakly inhibited CYP2C19-catalyzed [*S*]-mephenytoin 4′-hydroxylation and CYP2C8-catalyzed amodiaquine *N*-deethylation with IC_50_ values of 55.1 and 85.0 μM, respectively, without inhibition of CYP1A2-catalyzed phenacetin *O*-deethylation, CYP2A6-catalyzed coumarin 7-hydroxylation, CYP2B6-catalyzed bupropion hydroxylation, CYP2C9-catalyzed diclofenac 4′-hydroxylation, CYP2D6-catalyzed bufuralol 1′-hydroxylation, and CYP3A4-catalyzed midazolam 1′-hydroxylation activities at 100 μM in human liver microsomes (Figure 2).

**Figure 3:**
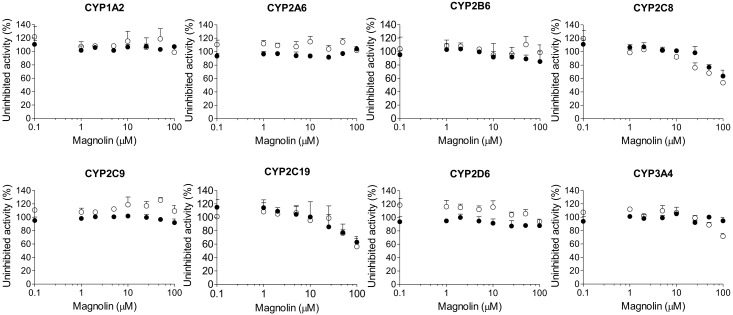
Inhibitory effects of magnolin on CYP1A2-mediated phenacetin *O*-deethylation, CYP2A6-mediated coumarin 7-hydroxylation, CYP2B6-mediated bupropion hydroxylation, CYP2C8-mediated amodiaquine *N*-deethylation, CYP2C9-mediated diclofenac 4′-hydroxylation, CYP2C19-mediated [*S*]-mephenytoin 4′-hydroxylation, CYP2D6-mediated bufuralol 1′-hydroxylation, and CYP3A4-mediated midazolam 1′-hydroxylation in pooled human liver microsomes. ○: Pre-incubation of liver microsomes with magnolin and NADPH for 30 min at 37 °C, ●: No pre-incubation. Data represent the average ± SD (*n* = 3).

**Figure 4:**
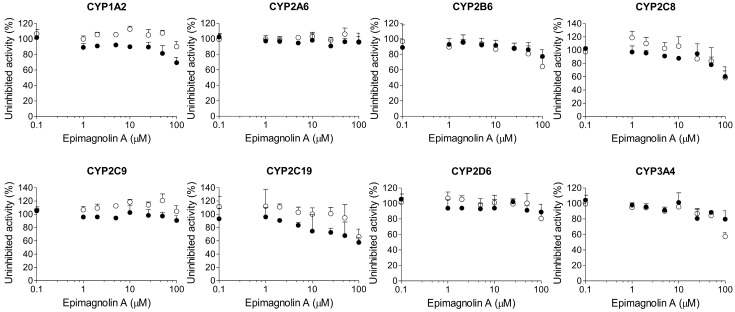
Inhibitory effects of epimagnolin A on CYP1A2-mediated phenacetin *O*-deethylation, CYP2A6-mediated coumarin 7-hydroxylation, CYP2B6-mediated bupropion hydroxylation, CYP2C8-mediated amodiaquine *N*-deethylation, CYP2C9-mediated diclofenac 4′-hydroxylation, CYP2C19-mediated [*S*]-mephenytoin 4′-hydroxylation, CYP2D6-mediated bufuralol 1′-hydroxylation, and CYP3A4-mediated midazolam 1′-hydroxylation in pooled human liver microsomes. ○: Pre-incubation of liver microsomes with epimagnolin A and NADPH for 30 min at 37 °C, ●: No pre-incubation. Data represent the average ± SD (*n* = 3).

**Figure 5:**
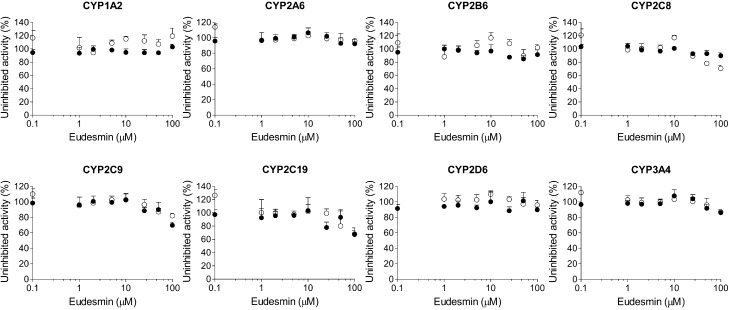
Inhibitory effects of eudesmin on CYP1A2-mediated phenacetin *O*-deethylation, CYP2A6-mediated coumarin 7-hydroxylation, CYP2B6-mediated bupropion hydroxylation, CYP2C8-mediated amodiaquine *N*-deethylation, CYP2C9-mediated diclofenac 4′-hydroxylation, CYP2C19-mediated [*S*]-mephenytoin 4′-hydroxylation, CYP2D6-mediated bufuralol 1′-hydroxylation, and CYP3A4-mediated midazolam 1′-hydroxylation in pooled human liver microsomes. ○: Pre-incubation of liver microsomes with eudesmin and NADPH for 30 min at 37 °C, ●: No pre-incubation. Data represent the average ± SD (*n* = 3).

Magnolin, epimagnolin A, and eudesmin negligibly inhibited CYP1A2, CYP2A6, CYP2B6, CYP2C8, CYP2C9, CYP2C19, CYP2D6, and CYP3A4 activities at 100 μM in human liver microsomes (Figure 3, Figure 4 and Figure 5).

**Figure 6:**
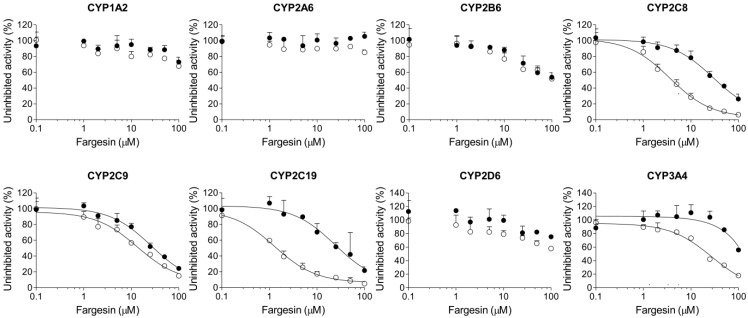
Inhibitory effects of fargesin on CYP1A2-mediated phenacetin *O*-deethylation, CYP2A6-mediated coumarin 7-hydroxylation, CYP2B6-mediated bupropion hydroxylation, CYP2C8-mediated amodiaquine *N*-deethylation, CYP2C9-mediated diclofenac 4′-hydroxylation, CYP2C19-mediated [*S*]-mephenytoin 4′-hydroxylation, CYP2D6-mediated bufuralol 1’-hydroxylation, and CYP3A4-mediated midazolam 1′-hydroxylation in pooled human liver microsomes. ○: Pre-incubation of liver microsomes with fargesin and NADPH for 30 min at 37 °C, ●: No pre-incubation. Data represent the average ± SD (*n* = 3).

Fargesin showed moderate inhibition of CYP2C8-mediated amodiaquine *N*-deethylation, CYP2C9-mediated diclofenac 4′-hydroxylation, and CYP2C19-mediated [*S*]-mephenytoin 4′-hydroxylation with IC_50_ values of 34.9, 30.8, and 30.2 μM, respectively, in human liver microsomes (Figure 6, Table 1). Fargesin at 100 μM showed negligible inhibition of CYP1A2, CYP2A6, CYP2B6, CYP2D6, and CYP3A4 activities in human liver microsomes (Figure 6).

A 30-min pre-incubation of dimethyllirioresinol, epimagnolin A, eudesmin, or magnolin with human liver microsomes and reduced β-nicotinamide adenine dinucleotide phosphate (NADPH) did not cause the IC_50_ value shift of eight CYP enzymes (Figure 2, Figure 3, Figure 4 and Figure 5), indicating that dimethyllirioresinol, magnolin, epimagnolin A, or eudesmin may not be mechanism-based inhibitors. However, 30 min pre-incubation of human liver microsomes with fargesin and NADPH lowered the IC_50_ values of CYP2C8-catalyzed amodiaquine *N*-deethylation, CYP2C19-catalyzed [*S*]-mephenytoin 4′-hydroxylation, and CYP3A4-catalyzed midazolam 1′-hydroxylation activities by more than 2.5-fold in comparison with the IC_50_ values obtained without pre-incubation (34.9 vs. 4.0 μM for CYP2C8, 30.2 vs. 1.6 μM for CYP2C19, and >100 vs. 17.9 μM for CYP3A4) (Figure 6, Table 1), indicating that fargesin causes potent mechanism-based inhibition of CYP2C8, CYP2C19, and CYP3A4 enzymes in human liver microsomes.

**Figure 7:**
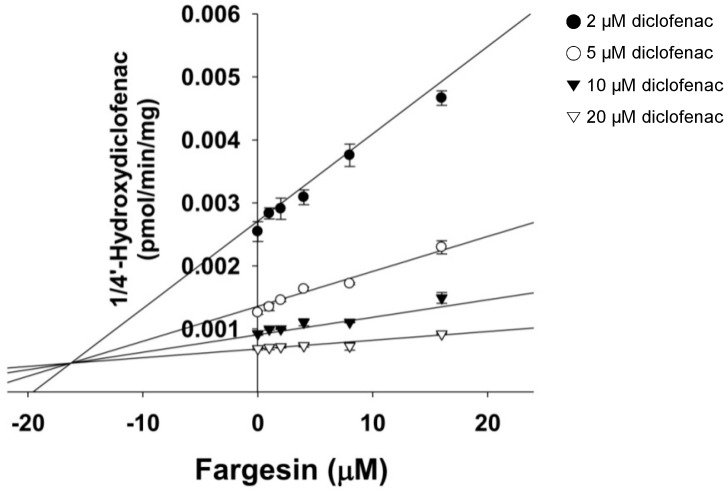
Dixon plot of the inhibitory effects of fargesin on CYP2C9-catalyzed diclofenac 4′-hydroxylation in pooled human liver microsomes. Data represent the average ± SD (*n* = 3).

**Figure 8:**
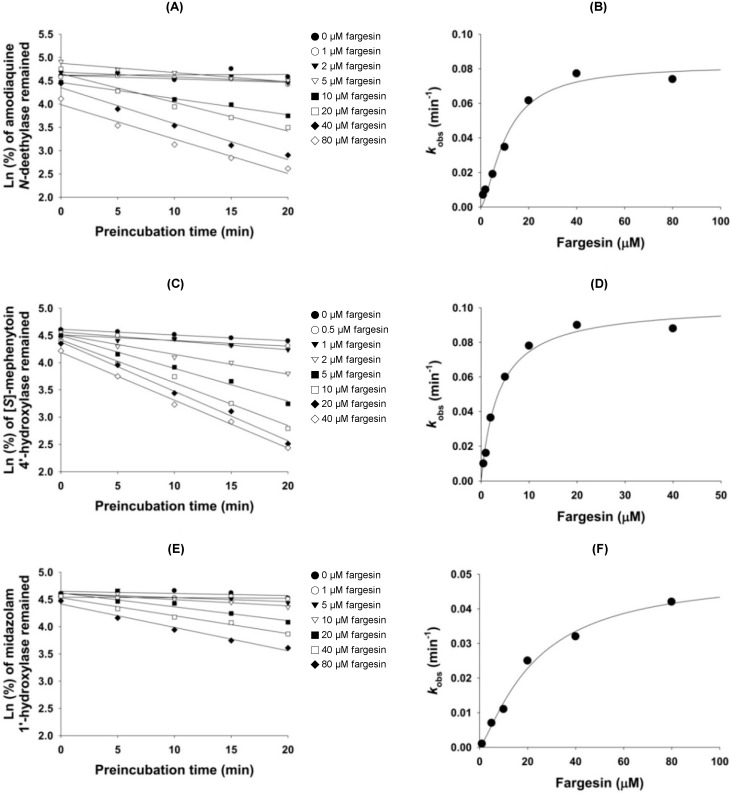
(**A**) Inactivation of human liver microsomal formation of *N*-desethylamodiaquine from amodiaquine by various fargesin concentrations; (**B**) The relationship between the observed *k* (*k*_obs_) and fargesin concentration for the estimation of the *K*_i_ and *k*_inact_ values of CYP2C8-catalyzed amodiaquine *N*-deethylation; (**C**) Inactivation of human liver microsomal formation of 4′-hydroxy-[*S*]-mephenytoin from [*S*]-mephenytoin by various fargesin concentrations; (**D**) The relationship between *k*_obs_ and fargesin concentration for the estimation of the *K*_i_ and *k*_inact_ values of CYP2C19-catalyzed [*S*]-mephenytoin 4′-hydroxylation; (**E**) Inactivation of human liver microsomal formation of 1′-hydroxymidazolam from midazolam by various fargesin concentrations; and (**F**) The relationship between the *k*_obs_ and fargesin concentration for the estimation of the *K*_i_ and *k*_inact_ values of CYP3A4-catalyzed midazolam 1′-hydroxylation.

In the study of enzyme inhibition, the inhibitor concentration causing half maximal inactivation (*K*_i_ value) and the inhibition mode define the interaction of an inhibitor with a particular enzyme. Fargesin exhibited competitive inhibition of CYP2C9-catalyzed diclofenac 4-hydroxylation with a *K*_i_ value of 16.3 μM (Figure 7, Table 1). Fargesin decreased CYP2C8-catalyzed amodiaquine *N*-deethylation, CYP2C19-catalyzed [*S*]-mephenytoin 4′-hydroxylation, and CYP3A4-catalyzed midazolam 1′-hydroxylation in pre-incubation time- and concentration-dependent manners in human liver microsomes (Figure 8). The apparent *K*_i_ and maximal inactivation rate (*k*_inact_) values of fargesin were 10.7 μM and 0.082 min^−1^ for CYP2C8-catalyzed amodiaquine *N*-deethylation, 3.7 μM and 0.102 min^−1^ for CYP2C19-catalyzed [*S*]-mephenytoin 4′-hydroxylation, and 23.0 μM and 0.050 min^−1^ for CYP3A4-catalyzed midazolam 1′-hydroxylation, respectively, in human liver microsomes (Table 1).

## 3. Discussion

In this study, the in vitro inhibitory effects of bioactive tetrahydrofurofuranoid lignans such as dimethyllirioresinol, epimagnolin A, eudesmin, fargesin, and magnolin on 8 major CYP enzymes were, for the first time, evaluated in pooled human liver microsomes. Dimethyllirioresinol exhibited weak inhibition of CYP2C8 and CYP2C19 activities without inhibition of CYP1A2, CYP2A6, CYP2B6, CYP2C9, CYP2D6, and CYP3A4 in human liver microsomes (Figure 2). Magnolin, epimagnolin A, and eudesmin showed no the reversible and time-dependent inhibition of CYP1A2, CYP2A6, CYP2B6, CYP2C8, CYP2C9, CYP2C19, CYP2D6, and CYP3A4 activities at 100 μM in human liver microsomes (Figure 3, Figure 4 and Figure 5). These results indicate that dimethyllirioresinol, epimagnolin A, eudesmin, and magnolin without a methylenedioxy ring in the chemical structure may not be CYP inhibitors in human liver microsomes. However, fargesin containing a methylenedioxyphenyl moiety in the chemical structure showed moderate reversible inhibition of CYP2C8, CYP2C9, and CYP2C19 activities (IC_50_ values of 34.9, 30.8, and 30.2 μM, respectively) and the potent time-dependent inhibition of CYP2C19, CYP2C8, and CYP3A4 activities (IC_50_ values of 1.6, 4.0, and 17.9 μM, respectively) in human liver microsomes. Aschantin, a chemical derivative of fargesin, with a methylenedioxyphenyl moiety also exhibited the reversible and time-dependent inhibition of CYP2C8, CYP2C9, CYP2C19, and CYP3A4 activities in human liver microsomes [32]. These results indicate that CYP inhibitory capacity of tetrahydrofurofuranoid lignans depends on the presence of a methylenedioxyphenyl moiety. Other methylenedioxyphenyl compounds such as myristicin and podophyllotoxin exhibited mechanism-based inactivation of CYP1A2 and CYP3A4, respectively, in human liver microsomes [36,37].

Fargesin exhibited competitive inhibition of CYP2C9-catalyzed diclofenac 4′-hydroxylation (*K*_i_, 16.3 μM), but aschantin showed mechanism-based inhibition of CYP2C9 (*K*_i_, 3.7 μM; *k*_inact_, 0.044 min^−1^) [32]. Other pharmacologically active lignans such as honokiol, deoxypodophyllotoxin, and podophyllotoxin potently inhibited CYP2C9 activity with *K*_i_ values of 0.54, 3.5, and 2.0 μM, respectively [33,36,38].

Fargesin showed mechanism-based inhibition of CYP2C8-catalyzed amodiaquine *N*-deethylation, CYP2C19-catalyzed [*S*]-mephenytoin 4′-hydroxylation, and CYP3A4-catalyzed midazolam 1′-hydroxylation in pooled human liver microsomes (Figure 8). The inactivation potency (*k*_inact_/*K*_i_ ratio) of fargesin against CYP2C8 (7.66 min^−1^ nM^−1^) was comparable to that of aschantin (*k*_inact_/*K*_i_ = 5.49 min^−1^ nM^−1^) [32], but was higher than those of mechanism-based CYP2C8 inhibitors such as amiodarone (0.57 min^−1^ nM^−1^), phenelzine (3.17 min^−1^ nM^−1^) [39], and gemfibrozil (1.24 min^−1^ nM^−1^) [40] in human liver microsomes, indicating that fargesin may be a potent mechanism-based inhibitor of CYP2C8.

The inactivation potency (*k*_inact_/*K*_i_ ratio) of fargesin against CYP2C19 (27.57 min^−1^ nM^−1^) was higher than those of drugs identified as mechanism-based inhibitors of CYP2C19 such as aschantin (8.28 min^−1^ nM^−1^), clopidogrel (3.90 min^−1^ nM^−1^), and fluoxetine (2.14 min^−1^ nM^−1^), but was comparable to that of ticlopidine (22.3 min^−1^ nM^−1^) [41] in human liver microsomes.

The CYP3A4 inactivation potency (*k*_inact_/*K*_i_ ratio) of fargesin (2.17 min^−1^ nM^−1^) was comparable with those reported for some phytochemicals identified as mechanism-based inhibitors of CYP3A4 including aschantin (4.92 min^−1^ nM^−1^) [32], bergamottin (2 min^−1^ nM^−1^) [42], and rutaecarpine (3.59 min^−1^ nM^−1^) [43], but much lower than those reported for podophyllotoxin (13.63 min^−1^ nM^−1^) [36], phyllanthin (131.88 min^−1^ nM^−1^) and hypophyllanthin (83.21 min^−1^ nM^−1^) [35] in human liver microsomes. The *k*_inact_/*K*_i_ ratio of fargesin against CYP3A4 was comparable to those of therapeutic drugs known as mechanism-based CYP3A4 inhibitors such as clarithromycin (1–13 min^−1^ nM^−1^), erythromycin (3–9 min^−1^ nM^−1^), amiodarone (4.5 min^−1^ nM^−1^), and fluoxetine (3.2 min^−1^ nM^−1^) in human liver microsomes [43].

## 4. Materials and Methods

### 4.1. Materials and Reagents

Epimagnolin A, eudesmin, fargesin, and magnolin were obtained from PhytoLab GmbH & Co. (Vestenbergsgreuth, Germany). Dimethyllirioresinol was a gift from Natural Medicine Research Center in Korea Research Institute of Biology and Biotechnology (Ochang, Korea). Bufuralol hydrochloride, 1′-hydroxybufuralol maleate, d_9_-1′-hydroxybufuralol maleate, bupropion, hydroxybupropion, 4′-hydroxydiclofenac, 1′-hydroxymidazolam, 4′-hydroxymephenytoin, [*S*]-mephenytoin, and pooled human liver microsomes (catalog number 452161) were purchased from Corning Life Sciences (Woburn, MA, USA). Amodiaquine hydrochloride, *N*-desethylamodiaquine dihydrochloride, acetaminophen, coumarin, 7-hydroxycoumarin, diclofenac sodium, midazolam, phenacetin, and NADPH were obtained from Sigma-Aldrich (St. Louis, MO, USA). ^13^C_2_, ^15^N-acetaminophen was obtained from Toronto Research Chemicals (Toronto, ON, Canada). Methanol, acetonitrile, and water (liquid chromatography-mass spectrometry [LC-MS] grade) were purchased from Fischer Scientific (Fair Lawn, NJ, USA). All other chemicals were of the highest quality available.

### 4.2. Inhibitory Effects of Dimethyllirioresinol, Epimagnolin A, Eudesmin, Fargesin, and Magnolin on 8 Major CYP Activities in Human Liver Microsomes

The degree of inhibition (IC_50_ values) of dimethyllirioresinol, epimagnolin A, eudesmin, fargesin, and magnolin toward CYP1A2, CYP2A6, CYP2C8, CYP2C9, CYP2C19, CYP2D6, and CYP3A4 activities in pooled human liver microsomes were evaluated following our previous method using CYP cocktail substrates and liquid chromatography-tandem mass spectrometry (LC-MS/MS) [33]. The incubation mixtures were prepared in total volumes of 100 μL as follows: 50 mM potassium phosphate buffer (pH 7.4), 1.0 mM NADPH, 10 mM MgCl_2_, pooled human liver microsomes (0.2 mg/mL), various concentrations of dimethyllirioresinol, epimagnolin A, eudesmin, fargesin, or magnolin in acetonitrile (final concentrations of 0.1–100 μM, acetonitrile 0.5% (*v*/*v*)), and a cocktail of seven CYP probe substrates (2.0 μM amodiaquine, 5 μM bufuralol, 2.5 μM coumarin, 10 μM diclofenac, 100 μM [*S*]-mephenytoin, 2.5 μM midazolam, and 50 μM phenacetin, acetonitrile 0.5% (*v*/*v*)). After 3 min pre-incubation at 37 °C, the reaction mixtures were incubated for 15 min at 37 °C with the addition of NADPH in a shaking water bath. The reaction was stopped by adding 100 μL of ice-cold methanol containing internal standards (d_9_-1′-hydroxybufuralol for 1′-hydroxybufuralol, 4′-hydroxydiclofenac, 7-hydroxycoumarin, 1′-hydroxymidazolam, and 4′-hydroxymephenytoin; ^13^C_2_, ^15^*N*-acetaminophen for acetaminophen and *N*-desethylamodiaquine). The mixtures were centrifuged at 13,000× *g* for 4 min at 4 °C. All assays were performed in triplicate and the average values were used for the subsequent calculations. For the measurement of time-dependent inhibition, human liver microsomes were pre-incubated with the various concentrations of dimethyllirioresinol, epimagnolin A, eudesmin, fargesin, or magnolin in acetonitrile (0.1–100 μM) and NADPH for 30 min at 37 °C. Then, the reaction mixtures were incubated with addition of the seven-CYP probe substrate cocktail for 15 min at 37 °C. The control reaction was performed by adding acetonitrile instead of the test compounds.

Seven metabolites were simultaneously determined using a tandem mass spectrometer (TSQ Quantum Access; Thermo Scientific, San Jose, CA, USA) equipped with an electrospray ionization (ESI) source coupled to a NANOSPACE SI-2 LC system (Shiseido, Tokyo, Japan). The column and autosampler temperatures were 50 and 6 °C, respectively. The ESI source settings for the ionization of metabolites were as follows: polarity, positive ion mode; capillary voltage, 4200 V; capillary temperature, 330 °C; vaporizer temperature, 350 °C; auxiliary gas pressure, 15 psi; and sheath gas pressure, 35 psi. Selected reaction monitoring (SRM) mode with the molecular ion and the intensive product ion was used for the quantification of each metabolite and internal standard, as follows: 1′-hydroxybufuralol, 278.1 > 186.1; *N*-desethylamodiaquine, 328.1 > 283.0; acetaminophen, 152.1 > 110.3; 7-hydroxycoumarin, 163.0 > 107.2; 4′-hydroxymephenytoin, 235.1 > 150.1; 4′-hydroxydiclofenac, 312.0 > 231.1; d_9_-1′-hydroxybufuralol, 287.2 > 187.0; and ^13^C_2_,^15^*N*-acetaminophen, 155.1 > 111.2. Analytical data were processed using Xcalibur™ software (Thermo Scientific, San Jose, CA, USA).

For the evaluation of the inhibitory effects of dimethyllirioresinol, epimagnolin A, eudesmin, fargesin, and magnolin on CYP2B6-catalyzed bupropion hydroxylation, each incubation mixture in a total volume of 100 μL contained 50 mM potassium phosphate buffer (pH 7.4), 10 mM MgCl_2_, pooled human liver microsomes (0.2 mg/mL), 50 μM bupropion, and various concentrations of dimethyllirioresinol, epimagnolin A, eudesmin, fargesin, or magnolin in acetonitrile (0.1–100 μM), according to our previous report [33]. After 3 min pre-incubation at 37 °C, the reaction mixtures were incubated with the addition of NADPH in a shaking water bath for 15 min at 37 °C. The reaction was stopped by adding 100 μL of ice-cold d_9_-1′-hydroxybufuralol (internal standard) in methanol. The mixtures were centrifuged at 13,000× *g* for 4 min at 4 °C. All incubations were performed in triplicate, and the average values were used for the subsequent calculations. For the measurement of time-dependent inhibition, pooled human liver microsomes were pre-incubated with various concentrations of dimethyllirioresinol, epimagnolin A, eudesmin, fargesin, or magnolin in acetonitrile (0.1–100 μM) and NADPH for 30 min at 37 °C. Then, the reaction mixtures were incubated with the addition of NADPH and bupropion for 15 min at 37 °C. The control reaction was performed by the addition of acetonitrile instead of the test compounds. Hydroxybupropion concentrations were quantified using the LC-MS/MS method described above; the SRM transitions for hydroxybupropion and d_9_-1′-hydroxybufuralol were 256.1 > 238.0 and 287.2 > 187.0, respectively.

### 4.3. Kinetic Analysis of CYP2C9 Inhibition by Fargesin

To determine the *K*_i_ values and inhibition mode of fargesin for CYP2C9, various concentrations of fargesin (0–16 μM) and diclofenac (2–20 μM) were incubated with human liver microsomes (0.1 mg/mL), 10 mM MgCl_2_, 1 mM NADPH, 50 mM potassium phosphate buffer (pH 7.4) in a total volume of 100 μL for 10 min at 37 °C. The reaction was stopped by adding 100 μL of ice-cold d_9_-1’-hydroxybufuralol in methanol (10 ng/mL), and the mixtures were centrifuged at 13,000× *g* for 4 min. 50 μL of the supernatant was diluted with 50 μL of water, and aliquots (5 μL) were analyzed by LC-MS/MS.

### 4.4. Mechanism-Based Inhibition of CYP2C8, CYP2C19, and CYP3A4 Activities by Fargesin

The mechanism-based inhibition potency of fargesin against human liver microsomal CYP2C8, CYP2C19, and CYP3A4 activities was evaluated. Human liver microsomes (1 mg/mL) were pre-incubated with various concentrations of fargesin and NADPH in 50 mM potassium phosphate buffer (pH 7.4). Aliquots (10 μL) of the pre-incubation mixtures were collected at 5, 10, 15, and 20 min after the pre-incubation and transferred to new tubes containing CYP substrates (2 μM amodiaquine for CYP2C8, 100 μM [*S*]-mephenytoin for CYP2C19, or 2 μM midazolam for CYP3A4), 10 mM MgCl_2_, 1 mM NADPH, and 50 mM potassium phosphate buffer (pH 7.4) in 90 μL reaction mixtures. The incubation was proceeded for 10 min and stopped by adding 100 μL of ice-cold d_9_-1′-hydroxybufuralol in methanol. The mixtures were centrifuged at 13,000× *g* for 4 min at 4 °C, and 50 μL of each supernatant was diluted with 50 μL of water. Aliquots (5 μL) were analyzed by LC-MS/MS, as described above.

### 4.5. Data Analysis

The IC_50_ values were calculated using SigmaPlot ver. 11.0 (Systat Software, Inc., San Jose, CA, USA). *K*_i_, *k*_inact_, and the inhibition mode were determined using Enzyme Kinetics ver. 1.1 (Systat Software, Inc.).

## 5. Conclusions

Fargesin competitively inhibited CYP2C9-catalyzed diclofenac 4′-hydroxylation with *K*_i_ value of 16.3 μM and exhibited the mechanism-based inhibition of CYP2C19-catalyzed [*S*]-mephenytoin 4′-hydroxylation, CYP2C8-catalyzed amodiaquine *N*-deethylation, and CYP3A4-catalyzed midazolam 1′-hydroxylation with *K*_i_ values of 3.7, 10.7, and 23.0 μM, respectively, in human liver microsomes. Fargesin negligibly inhibited CYP1A2, CYP2A6, CYP2B6, and CYP2D6 activities at 100 μM. Dimethyllirioresinol weakly inhibited CYP2C19 and CYP2C8 with IC_50_ values of 55.1 and 85.0 μM, respectively, without inhibition of CYP1A2, CYP2A6, CYP2B6, CYP2C9, CYP2D6, and CYP3A4 activities at 100 μM in human liver microsomes. Epimagnolin A, eudesmin, and magnolin showed no reversible or time-dependent inhibition of 8 major CYP activities at 100 μM in human liver microsomes. These in vitro results suggest that it is necessary to investigate fargesin-induced in vivo drug interaction studies via the inhibition of CYP2C8, CYP2C9, CYP2C19, and CYP3A4 activities.

## Figures and Tables

**Table 1:** Inhibitory effect of fargesin on eight major CYP enzyme activities in pooled human liver microsomes.

CYP	Enzyme Activities	IC_50_ (µM)		*K*_i_ (µM)
		No Pre-Incubation	With Pre-Incubation *	(*k*_inact_, min^−1^ or Inhibition Mode)
1A2	Phenacetin *O*-deethylase	>100	>100	-
2A6	Coumarin 7-hydroxylase	>100	>100	-
2B6	Bupropion hydroxylase	>100	>100	-
2C8	Amodiaquine *N*-deethylase	34.9	4.0	10.7 (*k*_inact_: 0.082)
2C9	Diclofenac 4′-hydroxylase	30.8	16.4	16.3 (competitive)
2C19	(*S*)-Mephenytoin 4′-hydroxylase	30.2	1.6	3.7 (*k*_inact_: 0.102)
2D6	Bufuralol 1′-hydroxylase	>100	>100	-
3A4	Midazolam 1′-hydroxylase	>100	17.9	23.0 (*k*_inact_: 0.050)

* 30 min pre-incubation of fargesin with microsomes and NADPH before the addition of CYP substrates. The substrate cocktail concentrations for the measurement of the IC_50_ values were as following: 50 μM phenacetin, 2.5 μM coumarin, 2.0 μM amodiaquine, 10 μM diclofenac, 100 μM [*S*]-mephenytoin, 5.0 μM bufuralol, and 2.5 μM midazolam. Inhibition of CYP2B6 activity was determined separately using 50 μM bupropion. The data represent the average of three determinations.
